# A network meta-analysis protocol of efficacy and safety evaluation of different surgery regimens for varicocele patients with infertility: A study protocol

**DOI:** 10.1097/MD.0000000000021150

**Published:** 2021-03-05

**Authors:** Xiao-dong Zhao, Xiao-ling Ma, Peng-cheng Ma, Jian-wen Wang

**Affiliations:** Department of Reproductive Medicine, The First Hospital of Lanzhou University, Lanzhou, China.

**Keywords:** efficacy evaluation, network meta-analysis protocol, varicocele, varicocelectomy

## Abstract

**Background:**

Surgical treatment of varicocele is still one of the most common important treatments for male infertility. Surgery regimens for varicocele (VC) is various, including high ligation, sub-inguinal, inguinal, retroperitoneal, laparoscopic, and microsurgery. The surgery regimens applied for VC patients are various in clinic, however, the significance, advantages, and disadvantages of different varicocelectomies for male infertility are still in controversial. Therefore, this network meta-analysis is mainly to assess the relative efficacy and safety of different surgery regimens for VC patients with infertility.

**Methods:**

To compare the relative efficacy and safety among different varicocelectomies for VC patients, we systematic searched randomized controlled trials (RCTs) and non-RCTs were in five electronic databases: Pubmed, Web of Science, EMBASE database, Clinical Trials, and Cochrane Library. Using R-3.4.1 software to process and analyze data. The bias risk of RCTs and non-RCTs will be evaluated through the tool of Cochrane Handbook version 5.1.0 and non-randomized studies of interventions (ROBINS-I), respectively.

**Results and conclusion:**

The result of this network meta-analysis aim is to evaluate the relative effectiveness and safety and rank the interventions among all surgery methods for VC patients and provide more evidence-based guidance in clinical practice.

**Protocol registration number:**

CRD42020162051.

## 1 Introduction

Varicocele (VC) is one of the common clinical diseases in andrology and is widely concerned for its related scrotal pain and discomfort, infertility and progressive testicular dysfunction, especially the impact on male fertility.^[1]^ VC is usually seen on the left side, accounting for 77% to 92%, bilaterally about 10% (7%–22%), and rare on the right side (<1%).^[2,3]^ Etiology of varicocele is complex and diverse, the present study suggests that may be associated with abnormal anatomy. In addition, there will be secondary to tumor compression, vascular tumor emboli or vascular compression of ectopic.^[4–6]^ Currently, surgery is the most effective treatment for male VC patients, and recent studies evidence indicated that early treatment of infertility patients due to varicocele would improve the long-term results, especially in testicular function and sperm function.^[7–9]^ The surgery regimens applied for VC patients in clinic are various, however, the significance, advantages and disadvantages of different varicocelectomies for male infertility are still in controversial.^[10]^ Therefore, this network meta-analysis is mainly to assess the relative efficacy of different surgery regimens for VC patients.

The drawback of paired meta-analysis is not being able to integrate all the information of different varicocelectomies from different original studies. Therefore, it is impossible to compare the pros and cons of different surgery regimens at the same time. The network meta-analysis can evaluate the relative effectiveness and rank the interventions among all surgery methods for VC patients, furthermore, it can also provide significant evidence-based guidance in clinical practice.^[11]^

The aim of this study is to evaluate the relative efficacy and safety of different surgery regimens for VC patients through this network meta-analysis.

## 2 Methods

### 2.1 Registration

The registration number was CRD42020162051. Our protocol has been registered on the international prospective register of systematic review (PROSPERO) network. This network meta-analysis protocol according to the preferred reporting items for systematic review and meta-analysis protocols (PRISMA-P) extension statement.^[12]^

### 2.2 Ethics and dissemination

#### 2.2.1 Ethics issues

The ethical approval or informed consent were not required in this meta-analysis, on account of this study is a secondary research based on published original data.

#### 2.2.2 Publication plan

This network meta-analysis is planned to be published in a peer-reviewed journal.

### 2.3 Inclusion criteria

#### 2.3.1 Types of studies

RCTs and non-RCTs are limited to trials involving adult patients with clinical infertility or at least 1 abnormal semen parameter and clinically detectable varicocele will be incorporated. The search deadline is December 12, 2019 and language limited to English.

#### 2.3.2 Types of participants

Adult male patients were confirmed as VC through imaging examination without racial and region limitations.

#### 2.3.3 Types of interventions

A total of six interventions: high ligation, sub-inguinal, inguinal, retroperitoneal, laparoscopic, and microsurgery. Based on the different surgery regimens in each intervention group, a more detailed subgroup analysis will be conducted to present the relative efficacy and safety.

#### 2.3.4 Type of outcomes

The primary outcomes are sperm parameters, sperm concentration, sperm motility, pregnancy rate and fertility. The secondary outcomes are complication, hospital stay time, and recurrence rate.

### 2.4 Information source

We systematic searched five databases which were listed as followings: PubMed, the Cochrane Library, EMBASE database, Web of Science, and Clinical Trials. We also manually retrieve the references in included studies as additional supplement.

### 2.5 Data collection and analysis

#### 2.5.1 Data management

Endnote X7 software will be used for literature managing and records searching. A pilot-test will be conducted to ensure the inter-rater is reliability between the reviewers before the literature selection.

#### 2.5.2 Selection process

Two experienced researchers will conduct a systematic search with the predetermined search strategy independently. In the case of the above-mentioned screening of documents and the extraction of data, any disagreement will be resolved by turning up to a third reviewer. The process of study selection will be indicated in a flow diagram in accordance with the PRISMA guidelines.^[13]^

#### 2.5.3 Data collection process

Two independent researchers extracted data in the same predetermined table through the excel software. Any disagreements will be resolved by a third reviewer. The extraction data items as following: the first author, country, published year, the trial design, sample size, age, surgery regimen, sperm parameters, sperm concentration, sperm motility, pregnancy rate, complication, hospital stay time, and recurrence rate of VC patients and some other outcomes of interest.

### 2.6 Quality of evidence assessment

According to Grading of Recommendations Assessment Development and Evaluation (GRADE), the quality assessment of included studies will be divided into high quality, moderate quality, low quality and very low quality through the online guideline development tool (GDT, http://gdt.guidelinedevelopment.org/).^[14]^

### 2.7 Risk of bias analysis

The risk of bias analysis of RCTs will be evaluated by the tool of Cochrane Handbook version 5.1.0 from seven specific domains (sequence generation, allocation concealment, blinding of participants and personnel, incomplete outcome data, selective reporting, and other bias and risk).^[15]^ According to the criteria of the risk of bias judgment, the methodological quality will be estimated as following: low risk, high risk, or unclear risk of bias.^[16]^ According to the tool for assessing risk of bias in non-randomized studies of interventions (ROBINS-I),^[17]^ the risk of bias of non-randomized studies will be estimated as following: confounding, the selection of participants, intervention classification, bias due to deviations from intended interventions, missing data, the measurement of outcomes, the selection of the result reporting, and overall risk bias. The risk of bias will be divided into five parts as following: low, moderate, serious, critical risk of bias, and no information. Two researchers perform the risk of bias assessment independently, any disagreements will be resolved by turning up to a third researcher.

### 2.8 Geometry of the network

The function of “forest.netmeta” of R-3.4.1 (R Foundation for Statistical Computing, Vienna, Austria) will be used to draw network plots to describe and present the geometry of different surgery regimens. The nodes and edges will be used to reveal the head-to-head comparisons among interventions.

### 2.9 Pairwise meta-analysis

The extracted data of all the included studies will be summarized and presented through Excel 2010. Pairwise meta-analysis will be conducted R-3.4.1 software. The statistical heterogeneity among included studies will be assessed by Higgins *I*^*2*^ statistic (large, if *I*^2^ > 50%; medium if 25% < *I*^2^ ≥ 50%; and small if 0 ≤ *I*^2^ ≥ 25%).^[18]^ Fixed-effect model analysis will be performed, if there is no evidence showed heterogeneity; otherwise, random-effect model analysis will be chosen after excluding the sources of heterogeneity.

### 2.10 Network meta-analysis

The “netmeta” version 0.9-8 of R-3.4.1 software will be used to perform a network meta-analysis to synthesize direct and indirect evidence for assessing the therapeutic effect and safety among different surgery regimens for VC adult patients.^[19]^ Inconsistency between direct and indirect comparisons will be assessed by the node splitting method when a loop connecting three arms existed. *P* scores will be used to rank the treatment effects of different surgery regimens for VC patients which are based on the point estimates and standard errors of the network assessment.

### 2.11 Other analyses

#### 2.11.1 Subgroup and sensitivity analyses

Subgroup analyses designed for age, region and different surgery regimens, which also could be used to find the possible sources of significant heterogeneity or inconsistency.

#### 2.11.2 Publication bias

STATA V.12.0 software will used to perform Egger's graph and Begg's graph to identify whether this meta-analysis will exist a publication bias.^[20]^

## 3 Discussion

This network meta-analysis is anticipated to provide significant evidence in surgery for VC patients with infertility.

## Author contributions

MXL and ZXD planed and designed the research; MPC and WJW tested the feasibility of the study; ZXD and MPC wrote the manuscript; all authors approved the final version of the manuscript.

**Conceptualization:** Xiaoling Ma, Xiaodong Zhao.

**Data curation:** Xiaodong Zhao.

**Investigation:** Jianwen Wang.

**Methodology:** Pengcheng Ma.

**Project administration:** Xiaoling Ma, Xiaodong Zhao.

**Resources:** Xiaoling Ma, Pengcheng Ma.

**Software:** Xiaodong Zhao.

**Supervision:** Jianwen Wang.
